# The cochlear basal turn as a very preserved region in cochlear hypoplasias: radiological and embryological considerations from a cohort of 125 patients

**DOI:** 10.1007/s00234-025-03671-5

**Published:** 2025-06-14

**Authors:** Antonio Messina, Emma Clement, Bernadette De Bakker, Robert Nash, Giovanni Morana, Shivaram Avula, Steve Connor, Felice D’Arco

**Affiliations:** 1 Paediatric Radiology Department, Our Lady’s Children’s Hospital, Dublin, Ireland; 2 Department of Clinical Genetics, Great Ormond Street Hospital for Children, London, United Kingdom; 3 Department of Obstetrics and Gynecology, Amsterdam UMC location University of Amsterdam, Meibergdreef 9, Amsterdam, The Netherlands; 4 Department of Medical Biology, Amsterdam UMC location University of Amsterdam, Meibergdreef 9, Amsterdam, The Netherlands; 5 Department of Paediatric Otolaryngology, Great Ormond Street Hospital, London, United Kingdom; 6 Neuroradiology Unit, Department of Neurosciences, University of Turin, Torino, Italy; 7 Department of Radiology, lder Hey Children’s NHS Foundation Trust, East Prescot Road, Liverpool, L14 5 AB United Kingdom; 8 School of Biomedical Engineering and Imaging Sciences, King’s College London , London, SE1 7EH United Kingdom; 9 Department of Neuroradiology, King’s College Hospital, London, SE5 9RS United Kingdom; 10 Department of Neuroradiology, Great Ormond Street Hospital for Children NHS Foundation Trust, London, WC1 N 3 JH United Kingdom

## Abstract

**Purpose:**

A distinct form of cochlear hypoplasia, characterized by the preservation of the first half of the basal turn with hypoplastic and anteriorly displaced upper turns, was historically associated with branchio-oto-renal (BOR) syndrome, but can also occur in other genetic, syndromic and non-syndromic causes of hearing loss. This study aims to describe this phenotype with relative preservation of the basal turn, particularly its first half, in a significant proportion of cochlear hypoplasia cases due to different causes.

**Methods:**

We retrospectively reviewed temporal bone imaging from 125 patients (250 ears) with cochlear malformations from a tertiary pediatric center, focusing on cases where the basal turn was partially or completely preserved. Temporal bone CT and internal auditory meatus MRI were assessed for cochlear morphology and associated anomalies and genetic, clinical and syndromic associations described.

**Results:**

Fifty-eight patients exhibited a preserved basal turn with different degrees of hypoplasia of the upper turns. These cases were grouped into five etiological clusters: branchio-oto-renal (BOR), CHARGE, Walker-Warburg (WWS) syndromes, other genetic cases and likely non-genetic cases (including syndromic conditions without a genetic cause identified such as oculo-auriculo-vertebral spectrum - OAVS). Genetic cases may show bilateral and symmetrical appearances, aberrant facial nerve courses were observed in 30 patients.

**Conclusions:**

Preservation of the first half of the basal turn suggests developmental arrest between 50 and 54 days of gestation, and is common across genetic and non-genetic conditions of cochlear hypoplasia. Frequent facial nerve anomalies may complicate cochlear implantation. Integrating imaging with embryological insights supports the need for refined, developmentally-based classification systems.

**Supplementary Information:**

The online version contains supplementary material available at https://doi.org/10.1007/s00234-025-03671-5.

## Introduction

Current classification schemes for inner ear malformations are largely based on morphology and aimed at guiding surgical planning [1]. Traditionally, these classifications link cochlear anomalies to specific gestational timing of developmental arrest, typically between the 3rd and 7 th week [2, 3]. However, in clinical practice, many cases do not fit neatly into a single category, or show features overlapping multiple types [4]. Moreover, recent embryological insights reveal that cochlear development extends into mid-gestation, further complicating strict phenotype-based classification [5].

A distinct cochlear hypoplasia phenotype—characterized by a preserved first half of the basal turn and hypoplastic, anteriorly displaced upper turns—has been described in BOR syndrome and linked to EYA1 variants [6–8]. While initially considered specific to BOR, similar features have been observed in other genetic and non-genetic syndromic conditions, including CHARGE, Walker-Warburg syndrome, and Oculo-Auriculo-Vertebral spectrum [9–11]. These overlapping phenotypes can, in many cases, be classified within both cochlear hypoplasia type 3 (CH3) and type 4 (CH4) of the Sennaroglu’s classification, without, however, precisely fitting in either of these groups [1].

The aim of this study is to characterize the relative preservation of the cochlear basal turn (in particular its first half) in a significant proportion of cases of cochlear hypoplasia. We also correlated this phenotype with the ex-vivo data from fetal studies, to get insights into the age of development of this specific malformation of the cochlear development.

This study also seeks to provide insights that may enhance the translation of embryological findings into surgical decision-making.

Precise characterization of cochlear malformations, such as the preservation of the basal turn, can influence pre-surgical planning, particularly in cochlear implantation where surgeons need detailed anatomical knowledge to anticipate challenges, such as electrode placement, and to maximize the likelihood of successful auditory outcomes.

## Materials and methods

From a large database of cochlear malformations of a single tertiary pediatric centre, including 125 patients (i.e. 250 ears) with clinical and genetic data (supplementary Table 1) we selected all the cases of cochlear hypoplasia where at least the first half of the basal turn was preserved.

Most genetic diagnoses were made using the deafness panel testing, except for Walker-Warburg syndrome (WWS), which was diagnosed through cerebral malformation panels. For syndromic conditions with non-genetic causes, such as oculo-auriculo-vertebral spectrum, clinical diagnosis was applied.

The basal turn of the cochlea was considered completely-developed when it displayed a full 360-degree rotation [12, 13].

A pediatric neuroradiologist with 10 years of experience in neuro-otology imaging and a pediatric neuroradiology fellow with 3 years of experience reviewed, in consensus, the medical records and imaging reports for cochlear anomalies identified on high-resolution temporal bone CT (thickness 0.5 mm) and/or 3D CISS (thickness 0.5 mm) on internal auditory meati (IAMs) 1.5 T and 3 T MRI scans, using both axial and coronal planes. Searches were conducted using specific keywords (Supplementary Material 2).

Cochlear height was measured using CT and/or MRI, which offer comparable accuracy [14], following reproducible techniques described in the literature [15, 16] and compared to a published threshold to exclude normal cochleae [17].

Imaging analysis:

For each case where, on at least one side, a cochlear malformation was confirmed, the following information was included:

- clinical presentation/syndromic diagnosis (if available);
- genetic abnormality (if available);
- types of diagnostic scans available (e.g. temporal bone petrous bone CT and/or, MRI IAMs);
- “cochlear height” measurement, as previously described;
- detailed description of the cochlear morphology;
- associated ear findings (depending on imaging availability);
- associated findings in other organ systems (depending on imaging availability).

We also added two cases of Pallister-Hall from other institutions, since the reported cochlear hypoplasia (when present) in these patients fit this phenotype [18].

Finally, we assessed the facial nerve canal using high-resolution temporal bone CT (0.5 mm).

3D CISS (0.5 mm) and 3D T1 MPRAGE (1 mm) obtained on 1.5 T and 3 T MRI scans were used to evaluate the cisternal and labyrinthine segments, respectively, in those cases when CT was not available.

## Results

We found 125 patients with at least one malformed cochlea, of those 58 showed on CT and/or MRI these findings on at least one side:

1. A relatively preserved basal turn of the cochlea,
2. Varying degrees of developmental abnormalities affecting the second half of the basal turn and the upper turns.

**Fig. 1:**
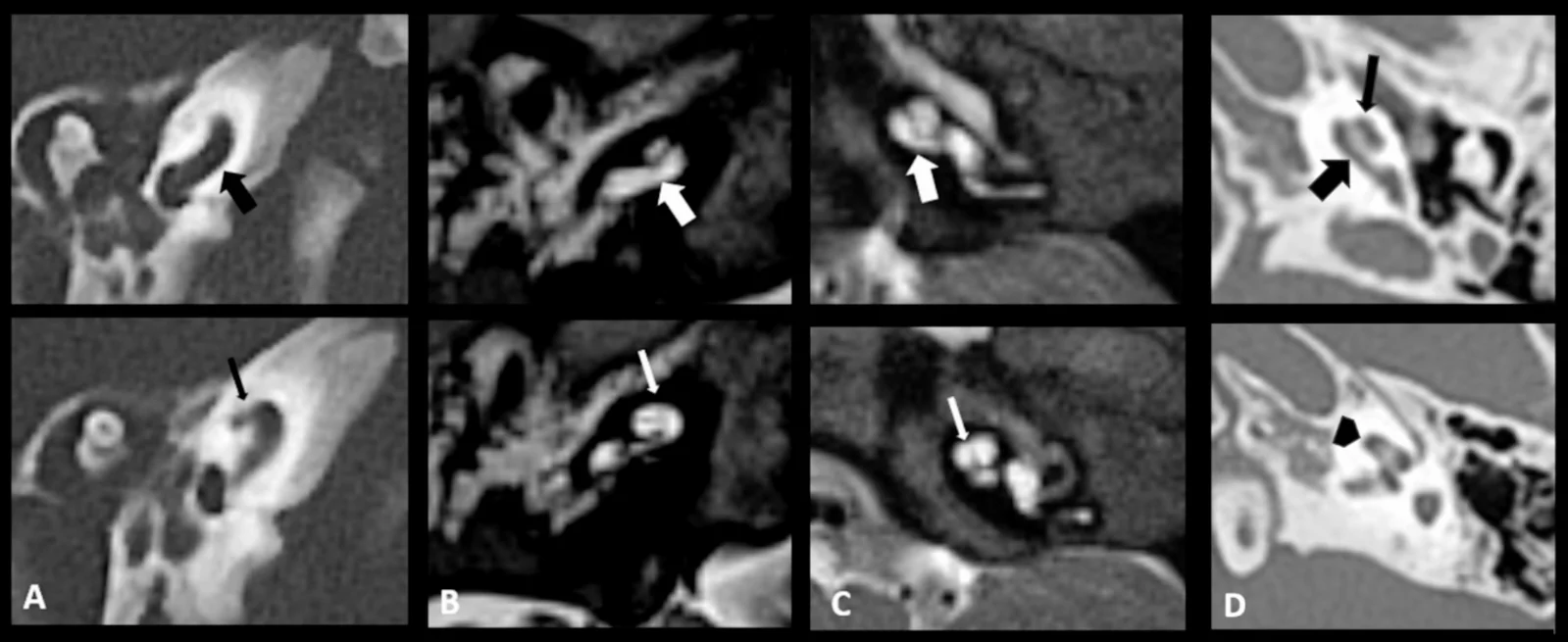
**(A)** Hypoplastic anterior off-set cochlea with preserved first half of the basal turn shown on an axial petrous bone CT in a patient with Walker Warburg syndrome due to a POMT1 gene mutation (A); the cochlea demonstrates a normal first half of the basal turn (thick arrow) whilst its second half and the upper turns are extremely hypoplastic and anteriorly off-set (thin arrow). **(B)** Axial 3D CISS in a patient with EYA1-BOR showing a normal first half of the basal turn (thick arrow), and moderately hypoplastic and anterior–off set second half and upper turns (thin arrow). **(C)** A patient with Goldenhar phenotype/oculo-auriculo-vertebral spectrum (OAVS), shows a hypoplastic cochlea with normal length of the basal turn (thick arrow) and hypoplastic and slightly anterior offset upper portions of the cochlea (thin arrow). **(D)** Finally, the mildest phenotype of cochlear hypoplasia with preserved basal turn is shown in a CHARGE patient with confirmed CHD7 mutation, where the first half of the basal turn is relatively preserved (thick arrow) but also the second half (arrowhead) is present and fully developed (although slightly rotated anteriorly); conversely, the upper turns are small and dysplastic (thin arrow). In cases A, B, and D the cochlear findings were symmetrical, while in case C the contralateral cochlea was nearly normal with only subtle hypoplasia of the modiolus

This phenotypic spectrum, termed cochlear hypoplasia with preserved basal turn (CHPB), was associated with both genetic and non-genetic etiologies. We categorized the patients into five distinct etiology clusters: three representing the most common cochlear syndromes in our population: BOR, CHARGE, and Walker-Warburg (WWS) syndrome (the latter 2 groups including confirmed cases from 2 previously published case series [9, 10]); one comprising patients with other confirmed genetic abnormalities (some in a syndromic context); and a final group including patients with likely non-genetic or unknown etiologies that did not fit into the other categories, including syndromic conditions without a genetic cause identified such as oculo-auriculo-vertebral spectrum - OAVS (Fig. 1; Table 1 and supplementary Table 1).

**Table 1:** Radiological findings in patients categorized as different etiology clusters, with at least one preserved first half of the basal turn

Etiology clusters	*n*° patients	*n*° cochleae with CHPB*	*n*° cochleae with only first half basal turn - phenotype	*n*° cochleae with complete 360° basal turn - phenotype	Bilateral cases	Symmetric cases	Asymmetric cases	Aberrant VII nerve cases
CHARGE	21	36	10	26	17	13	4	2
BOR	15	23	23	0	11	11	0	11
Walker- Warburg	6	12	12	0	6	6	0	6
Other genetic	31	16	13	3	7	6	1	3
Unknown/ likely non-genetic	52	19	9	10	8	6	2	8
**Total**	**125 ** **patients**	**106** **cochleae**	**67 ** **cochleae**	**39 ** **cochleae**	**50** **cases**	**43** **cases**	**7** **cases**	**30** **cases**

*CHPB: cochlear hypoplasia with preserved basal turn

This was also, by far, the most common form of cochlear hypoplasia found in our population with only 6 other cochlear hypoplasias not fitting the aforementioned description: 4 flattened cochleae with all turns preserved related to SOX10 mutation and 2 with a cochlear bud (cochlear hypoplasia type 1) (see supplementary Table 1).

Out of the 15 patients diagnosed with BOR syndrome, 12 presented with a preserved first half of the basal turn and a moderately hypoplastic second half, as well as middle and apical turns. The EYA1 gene was the most commonly mutated, and the majority (11/12) displayed bilateral and symmetrical findings.

In contrast, among patients with CHARGE syndrome (confirmed genetically and/or with fitting the clinical diagnostic criteria [19]), 7 exhibited a preserved first half of the basal turn, while 16 showed a well-developed basal turn. Bilateral and symmetrical patterns were noted in 13 patients.

The 6 patients with WWS (as previously reported [10]) had a normal first half of the basal turn associated to a markedly hypoplastic and anteromedially displaced upper parts of the cochlea (similar but more pronounced than the BOR phenotype) in a bilateral and symmetrical fashion (6/6).

Among the 31 patients with cochlear malformations due to other genetic causes (see supplementary Table 1), 7 exhibited only a preserved first half of the basal turn, while 2 showed a completely well-developed basal turn.

Finally, within the broader group of patients with likely non-genetic and/or unknown causes of cochlear abnormalities, 6 had a preserved first half of the basal turn, while 7 exhibited a well-developed basal turn.

Additionally, among the 58 patients with this cochlear phenotype, 30 exhibited an aberrant course of the facial nerve or canal. This presented either as a mild anterior displacement of the labyrinthine segment (type 2a according to Sennaroglu et al. [20]), seen in 25 cases, or as the cisternal segment coursing anteriorly towards the Gasserian ganglion in an enlarged Meckel’s cave in children with Goldenhar phenotype/oculo-auriculo-vertebral spectrum (OAVS) which has been previously described in patients with OAVS and recently linked to maternal diabetes [21, 22].

## Discussion

Although the molecular factors involved in the development of the otic capsule are still poorly understood [23], it is known that around 34 to 41 development days (five weeks of gestation), the cochlea begins to develop from an elongated otocyst.

The first half of the basal turn likely develops around between 44 and 54 days (around 7 th-8 th week of gestation), showing progressive changes in its orientation as it follows the surrounding petrous bone [24].

By the 54 th day, the complete basal turn should be present, and the entire labyrinth is expected to develop by the 60 th day [5].

**Fig. 2:**
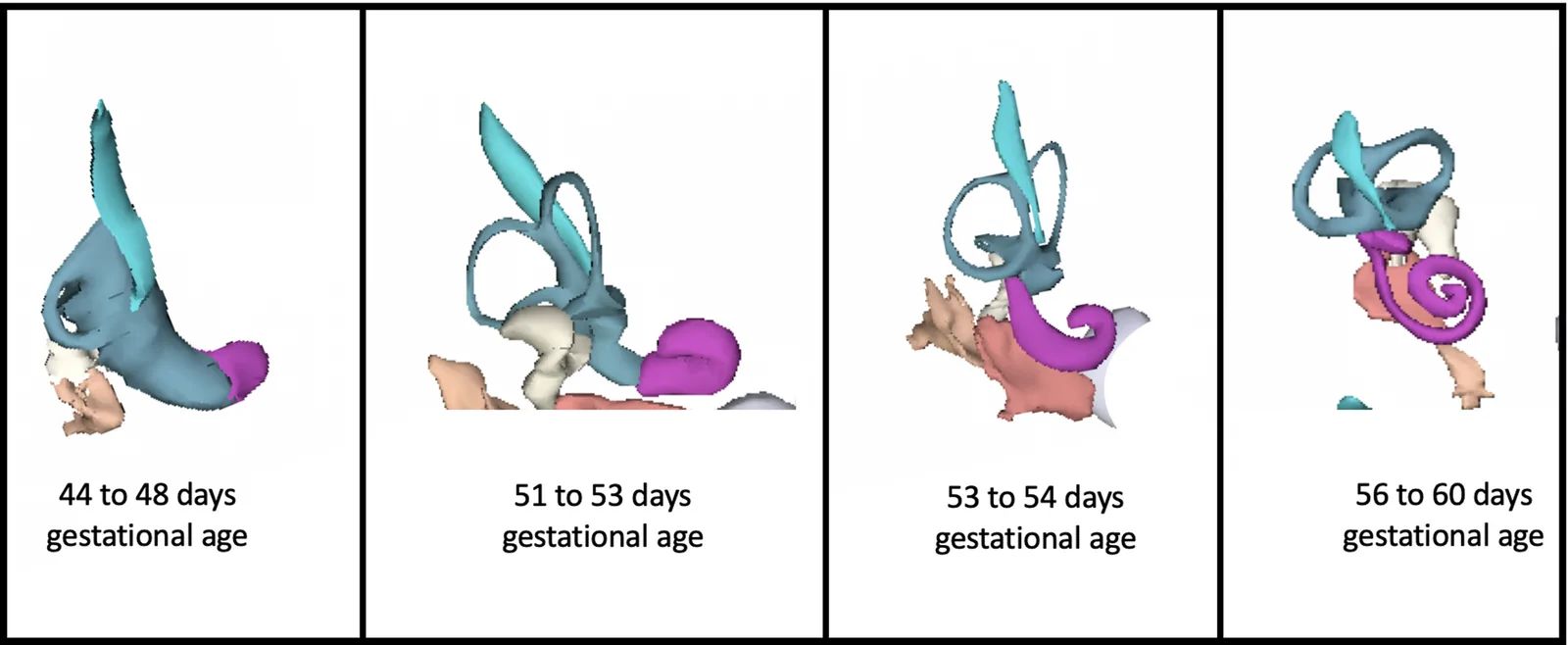
Ex-vivo reformats of the inner ear between 44 and 60 days (approximately 7 th to 10 th week of gestation) of developmental age (from *3 dembryoatlas.com*) showing the basal turn development. This time window seems to be critical, since the described phenotype of cochlear hypoplasia appears very similar to the ex-vivo reformats between the 44th and 54th day. Purple: cochlea. Grey-blue: semicircular canals. Light blue: vestibular aqueduct

Using the 3D atlas of human embryology created by the University of Amsterdam (*3 dembryoatlas.com*), ex vivo radiological images of the cochlea demonstrate a developmental pattern consistent with embryological descriptions and our radiological findings (Fig. 2).

Specifically, during the formation of the first half of the basal turn, the remaining cochlear structures appear displaced anteriorly, similar to the radiological description of an “unwound” cochlea.

Therefore, the period between the 44 th and 54 th day appears to be crucial for the development of the cochlear basal turn and likely represents a critical point in time when developmental arrest occurs, leading to the majority of cases of cochlear hypoplasia. The spectrum of cochlear hypoplasias we described probably reflects different ages of developmental arrests between this 10-day time interval.

Particularly, the phenotype in WWS, represents the most severe manifestation of this spectrum, with a normal first half of the basal turn and extreme hypoplastic second half and upper turns which appear anteriorly off-set (Fig. 1A).

BOR syndrome associated with EYA1 mutations, together with other syndromic or genetic causes cochlear hypoplasia, exhibit an intermediate phenotype: where the basal turn shows again a normal first half, while the second half and the upper turns are moderately hypoplastic and anteriorly offset, though less prominently than in the WWS phenotype (Fig. 1B and C).

A similar appearance is described and classified as a “moderate phenotype” in the CHARGE syndrome spectrum, showing a normal first half associated with hypoplastic anteriorly off-set second half/upper turns [9].

Finally, in CHARGE and other conditions, a most common “mild phenotype” is observed where the basal turn is fully developed, while the upper turns exhibit varying degrees of hypoplasia and, conversely, are less anteriorly displaced (Fig. 1D) [9].

The current classification system groups cochlear hypoplasias into four categories [1], but recent advances in embryology, genetics, and syndromic correlations reveal significant overlap and variability within these phenotypes. In particular, cochlear hypoplasia type 4 (CH4) shows a continuum of abnormalities with varying degrees and patterns of basal turn development.

Our findings emphasize that the first half of the cochlear basal turn is a well-preserved structure and suggest that the “anterior offset” appearance is merely a result of an arrest at a specific developmental stage of the cochlea. This would further explain why the upper turns hypoplasia is more pronounced in conditions where this feature (i.e. “anterior offset”) is more accentuated, such as in Walker-Warburg syndrome. This phenotype (i.e. CHPB) is observed in both genetic and non-genetic cases, highlighting the critical role of the timing of the developmental insult, rather than exclusively the specific nature of the insult itself.

Additionally, within our cohort, this phenotype was strongly associated with an abnormal course of the facial nerve canal. Interestingly, we observed that among the 25 cases with anterior displacement of the facial labyrinthine segment [20], 24 were associated with only a preserved first half of the basal turn while only one with a fully developed basal turn. This observation aligns with previous studies [25, 26], suggesting that the facial nerve canal location depends on the anatomy and size of the otic capsule, which, in turn, are abnormal in cochlear hypoplasias. Increased surgical challenges in patients with abnormal facial nerve anatomy and cochlear malformations have been reported, thus this observation is important for pre-surgical planning [13, 27].

The literature also includes cases where the facial recess is abnormally oriented in relation to the basal turn of the cochlea, emphasizing its role in determining the correct surgical approach [28, 29].

These studies demonstrate the importance of investigating and reporting the course of the facial nerve in patients with cochlear hypoplasia, to provide the surgeon with an accurate preoperative anatomical assessment, guiding the selection of the most appropriate surgical approach [30].

Another essential aspect of ear development is the polarization of specific genes, which can predominantly influence either the anterior (cochlear) or posterior (vestibular and semicircular canals) regions of the developing ear. The specific genetic mutations involved may, thus, help predict the resulting phenotype, and vice versa [23, 31]. Mutations associated with CHARGE syndrome often result in severe malformations of the posterior otic capsule, with total or near-total absence of the semicircular canals, while the cochlea can be relatively preserved. In contrast, WWS primarily affects the anterior otic capsule, leading to a severely abnormal cochlea while sparing the semicircular canals. These findings, combined with prior genotype-phenotype correlations, offer significant potential for identifying new genes involved in ear development and understanding their specific roles and functions [4, 10].

 For example, many genes, including SIX family members and EYA1, are known to play crucial roles in various stages of ear and kidney development, maintaining progenitor populations, forming specialized cell types, and balancing proliferation and differentiation in otic neurons and nephron progenitors [32]. Interestingly, these two organs seem to share a similar critical late embryonic window. The nephron, the structural and functional unit of the kidney, first appears around the 44 th to 49 th day of gestation and gradually establishes connections with the urinary collecting system [33]. This developmental overlap may help explain the similarities in their ultrastructural appearance, physiological functions, and certain pharmacological responses [34]. Furthermore, it provides insight into why many genes involved in this cochlear hypoplasia spectrum phenotype, as seen in syndromes such as BOR, CHARGE, Walker-Warburg, Pallister-Hall, and Williams-Beuren, are often associated with renal abnormalities [4, 35–37].

This study is based on data from a single tertiary pediatric institution, which may limit the generalizability of the findings to other populations. While the sample size is substantial, the cohort may be subjected to referral biases, as patients with more complex or rare cochlear malformations may be overrepresented. Diagnostic biases might also influence the phenotypic distribution, as the study was conducted at a specialized center with expertise in cochlear malformations. Furthermore, the retrospective nature of the study restricts our ability to draw definitive conclusions regarding causal relationships.

We do recognise the need for an updated cochlear malformation classification system, to be validated through multicenter analysis to ensure robustness and broader applicability of the findings.

## Conclusion

This study emphasizes that the cochlear basal turn (especially its first half) is a well-preserved structure in most cases of cochlear hypoplasia. Our findings suggest that a specific timing of the developmental arrest (between the 44th and 54th day) plays a critical role in this, which appears to be the most common type of cochlear hypoplasia, which can be found in both genetic cases and cases that are likely non-genetic. Additionally, we observed a correlation between this CHPB phenotype and abnormal facial nerve anatomy, which carries important implications for surgical planning, particularly in cochlear implantation.

By combining imaging findings with ex-vivo embryological data, we provide new insights into cochlear development and stress the need for updated classification systems that capture the full spectrum of phenotypic variations. To validate these observations and deepen our knowledge about the complexity of inner ear malformations, prospective multicentre studies, including larger and diverse patient cohorts, will be essential.

## Electronic supplementary material

Below is the link to the electronic supplementary material.

Supplementary Material 1 (XLSX 252 KB)

Supplementary Material 2 (DOCX 6.80 KB)

Supplementary Material 3 (PNG 2.87 MB)

## Data Availability

No datasets were generated or analysed during the current study.

## Declarations

### **Data transparency**

The authors confirm that all data, materials, and software applications or custom code supporting the published claims are available in accordance with field standards and the journal's data sharing policy.
