# Supplementary material for: The cochlear basal turn as a very preserved region in cochlear hypoplasias: radiological and embryological considerations from a cohort of 125 patients
Source: Neuroradiology. 2025 Jun 14;67(7):1945–52. doi: 10.1007/s00234-025-03671-5 (PMC12390867; doi:10.1007/s00234-025-03671-5)
Supplement: Supplementary file 2 — Supplementary Material 2 (DOCX 6.80 KB) [file 234_2025_3671_MOESM2_ESM.docx]

*Supplementary material 2: keywords for patient selection*

*- cochlea dysplasia*

*- cochlea hypoplasia*

*- IP or incomplete partition*

*- Mondini or Mondini triad or Mondini malformation*

*- cochlear aplasia or aplastic cochlea*

*- X-linked deafness*

*- Pendred or Pendred Syndrome*

*- EVA*

*- enlarged vestibular aqueduct*

*- cochlear bud*

*- common cavity*

*- BOR or Branchio-oto-renal syndrome;*

*- SOX10*

*- Waardenburg syndrome*

*- LVA*

*- LVAS*

*- LESA*

*- cystic cochleovestibular anomaly*

*- CHARGE syndrome*

*- Goldenhar syndrome/ oculo-auriculo-vertebral spectrum/ hemifacial microsomia*
