# Supplementary figures and images for: The cochlear basal turn as a very preserved region in cochlear hypoplasias: radiological and embryological considerations from a cohort of 125 patients
Source: Neuroradiology. 2025 Jun 14;67(7):1945–52. doi: 10.1007/s00234-025-03671-5 (PMC12390867; doi:10.1007/s00234-025-03671-5)

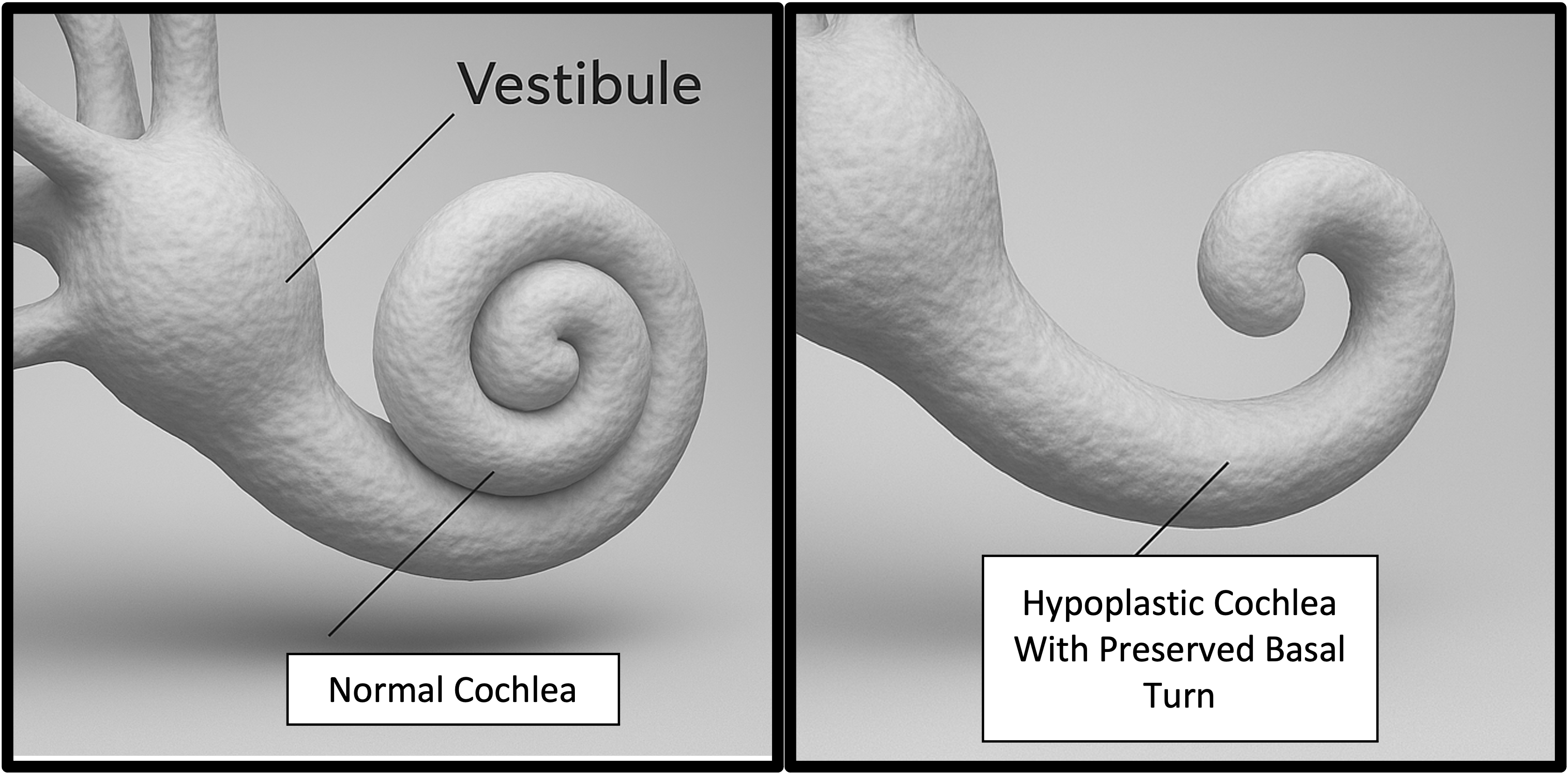

Supplement: Supplementary file 3 — Supplementary Material 3 (PNG 2.87 MB) [file 234_2025_3671_MOESM3_ESM.png]
